# Granulocyte differentiation of rat bone marrow resident C-kit^+^ hematopoietic stem cells induced by mesenchymal stem cells could be considered as new option in cell-based therapy

**DOI:** 10.1016/j.reth.2023.04.004

**Published:** 2023-05-03

**Authors:** Raheleh Farahzadi, Ezzatollah Fathi, Seyed Alireza Mesbah-Namin, Ilja Vietor

**Affiliations:** a Hematology and Oncology Research Center, Tabriz University of Medical Sciences, Tabriz, Iran; b Department of Clinical Sciences, Faculty of Veterinary Medicine, University of Tabriz, Tabriz, Iran; c Department of Clinical Biochemistry, Faculty of Medical Sciences, Tarbiat Modares University, Tehran, Iran; d Institute of Cell Biology, Medical University of Innsbruck, Biocenter, Innsbruck, Austria

**Keywords:** Mesenchymal stem cells, Granulocyte differentiation, Rat BM-Resident C-kit^+^ HSCs, Cell based-therapy, Clinical agent

## Abstract

Mesenchymal stem cells (MSCs) are effective in hematopoietic engraftment and tissue repair in stem cell transplantation. In addition, these cells control the process of hematopoiesis by secreting growth factors and cytokines. The aim of the present study is to investigate the effect of rat bone marrow (BM)-derived MSCs on the granulocyte differentiation of rat BM-resident C-kit^+^ hematopoietic stem cells (HSCs). The mononuclear cells were collected from rat BM using density gradient centrifugation and MSCs and C-kit^+^ HSCs were isolated. Then, cells were divided into two groups and differentiated into granulocytes; C-kit^+^ HSCs alone (control group) and co-cultured C-kit^+^ HSCs with MSCs (experimental group). Subsequently, the granulocyte-differentiated cells were collected and subjected to real-time PCR and Western blotting for the assessment of their telomere length (TL) and protein expressions, respectively. Afterwards, culture medium was collected to measure cytokine levels. CD34, CD16, CD11b, and CD18 granulocyte markers expression levels were significantly increased in the experimental group compared to the control group. A significant change was also observed in the protein expression of Wnt and β-catenin. In addition, MSCs caused an increase in the TL of granulocyte-differentiated cells. MSCs could affect the granulocyte differentiation of C-kit^+^ HSCs via increasing TL and Wnt/β-catenin protein expression.

## 1 Introduction

Mesenchymal stem cells (MSCs) are heterogeneous subset of stromal cells. They can differentiate into multi-lineage cells like osteocytes, adipocytes, chondrocytes etc. [1]. These cells have the ability to isolate from different tissues including adipose, amniotic fluid, bone marrow (BM), heart, liver, peripheral blood, umbilical cord blood (UCB), etc. [2,3]. BM is an important source of MSCs for experimental purposes. BM microenvironment contains also hematopoietic stem cells (HSCs), epithelial cells (EPCs), and blood cells in various stages of differentiation [4]. HSCs grow in the BM cavity and remain there until they reach maturity and are released into the vascular system. MSCs as well as stromal cells play a key role in organizing and formation of the specialized microenvironment of the BM. MSCs derived from BM present a suitable complex network of cytokines, scaffold, extracellular matrix proteins, and adhesion molecules. They interact with HSCs and offer a physical support for differentiation and maturation of these through the secretion of cytokines [5]. The studies show that MSCs promote HSCs expansion in the *in vitro* situation [6,7]. A study by Silva et al. (2005), indicated that human stromal cells in the presence of proper cytokines promote the *ex vivo* expansion of BM and CB-resident CD34^+^ HSCs [8]. Li et al. (2007) showed that MSCs efficiently improve *ex vivo* expansion and decrease stimulatory capacity of peripheral blood CD34^+^ progenitor cells (PCs), respectively [6]. Lazarus et al. in 2005, reported firstly that after myeloablative therapy, co-transplantation of MSCs and HSCs from HLA-identical sibling donors could lessen graft-versus-host disease (GVHD) [9]. MSCs have beneficial effects on engraftment due to their supportive role in hematopoiesis. In regenerative medicine, MSCs can be expanded *in vitro* for healing of damaged tissues [[10], [11], [12], [13]]. It was shown that these cells repair damaged tissues through differentiation of residing tissues. Therefore, stem cells as a therapeutic approach are considered in the treatment of many diseases [14]. However, the role of different cell types in the BM niche in the differentiation of HSCs is not known.

**Table 1:** Primer sequences used in absolute telomere length assay.

Oligomer name	Primer pair sequence (5′-3′)	PCR product size (bp)
**Telomere standard**	(TTAGGG)14	84
**36B4 standard**	5′CCTTGTCTCCAGTCTTTATCAGCTGCACATCGCTCTGAGGA AGAGAAGAGCAGTTACCACCCAGACACACAGAAG 3′	75
**Telo**	Fwd: CGGTTTGTTTGGGTTTGGGTTTGGGTTTGGG TTTGGGTT Rev: GGCTTGCCTTACCCTTACCCTTACCC TTACCCTTACCCT	>76
**36B4**	Fwd: CTTCTGTGTGTCTGGGTGGT Rev: CCTTGTCTCCAGTCTTTATCAG	75

Previous studies demonstrated that MSCs enhance the differentiation of hematopoietic PCs toward myeloid and lymphoid lineages [15]. Chen et al. (2013) showed that UCB-derived MSCs are capable of inducing granulocytic differentiation of both, primary acute promyelocytic leukemia (APL) and the APL-derived NB4 cells [16]. Next, Nikkhah et al. (2018) reported that the granulocyte differentiation of human promyelocytic leukemia cell line HL-60 could be promoted by BM derived-MSCs [17]. Additionally, this effect was found to be associated with the increased CD11b cell surface marker expression [17].

The role of different signals as developmental regulators in maturation of HSCs, e.g. Wnt and β-catenin pathway that regulates proliferation, differentiation, apoptosis, and aging of HSCs was previously reported [18]. The role of the Wnt family proteins in HSCs development was subject of several studies. Wnt signaling regulates HSCs self-renewal, BM recovery and repopulation [19] or that the activation of Wnt pathway increased the number of HSCs [20]. On the other hand, the inhibition of Wnt signaling decreases hematopoietic output [21]. Regulation of HSCs differentiation and proliferation are mediated by many factors, e.g., interleukins and colony stimulating factors.

Importantly, *in vitro* expansion of HSCs for cell transplantation is the preferred topic of investigation. Hematopoiesis can be defined as the interaction of hematopoietic and stromal cells in the BM microenvironment. The BM microenvironment is derived from common progenitors of mesenchymal origin. The role of different cell types of the BM microenvironment in the differentiation potentiation of HSCs is yet to be understood. Particularly, MSCs, as precursors of cellular components have an effective role in differentiation of HSCs. Co-transplantation of MSCs and HSCs promoted the engraftment of HSCs [22].

Studies of MSCs effects on HSCs differentiation can extend solutions in the modern treatment of patients with hematologic malignancies, mainly to increase the benefit of BM transplantation.

This study intends to investigate the impact of co-culturing of MSCs on the differentiation of rat BM-resident C-kit^+^ HSCs into the granulocytes. Furthermore, the Wnt and β-catenin protein expressions, as the key component of signaling pathways in the hematopoiesis, were investigated. Next, the influence of MSCs on granulocyte differentiation potential of rat BM resident C-kit^+^ HSCs was evaluated based on the absolute telomere length and cytokines measurements.

## 2 Material and methods

### 2.1 Isolation of rat BM-derived MSCs

BM mononuclear cells (MNCs) were collected using density gradient centrifugation as previously described by Fathi et al. (2022) [23]. For this purpose, the ethical code was obtained from the Committee on the Ethics of Tabriz University of Medical Sciences (EthicsNo. IR. TBZMED.VCR.REC.1397.322). Five males Rattus Norvegicus were euthanized. Then, BM was flushed and layered over the Ficoll-Paque (Innotrain, Germany). The obtained content was centrifuged at 850×*g* for 25 min at 4 °C. MNCs were seeded in 6-well plates (SPL Life Sciences Co., Pocheon, Korea) containing DMEM low glucose supplemented with 10% FBS (Gibco Co., Carlsbad, USA). Cells were cultured until they reached 70–80% confluence.

### 2.2 Characterization of rat BM-derived MSCs

MSCs were characterized using multi-lineage differentiation and investigation of cell surface markers by flow cytometry. In brief, rat BM-MSCs were cultured in adipogenic and osteogenic induction medium (Gibco Co., Carlsbad, USA). At the end of day 21, the cells were stained with Oil red O (Idea Zist, Iran) and Alizarin red (Idea Zist, Iran) for adipogenesis and osteogenesis assessment, respectively [24]. Also, flow cytometry method was used for immunophenotypical characterization of CD31, CD34, CD44, and CD73 [25]. To accomplish this, 1 × 10^6^ BM-MSCs/well at passage 4 were harvested and stained with antibodies against CD31, CD34, CD44, and CD73 with incubation of 30 min at 4°. Following the staining process, the cells were washed with PBS containing 3–5% FBS and analyzed by FACS for the determination of cell surface markers.

### 2.3 Isolation of rat BM-resident C-kit^+^ HSCs

The magnetic-activated cell sorting (MACS) technique was used to isolate and enrich CD44^+^ cells. For isolating the rat BM-resident C-kit^+^ HSCs, MNCs obtained from the previous step were incubated with C-kit^+^ micro beads (Miltenyi Biotec, Germany; Cat no: 130–091–224) for 30 min at 4 °C on a rotator. Then, the cells were washed with PBS containing FBS (0.5%), passed through one LS MACS column (Miltenyi Biotec, Germany) and enriched C-kit^+^ cells were retrieved by flushing the column. At the end of separation, C-kit^+^ cells were harvested as HSCs for subsequent analysis.

### 2.4 Characterization of rat BM-resident C-kit^+^ HSCs

Flow cytometry was used for purity assessment of enriched C-kit^+^ HSCs. In brief, 20 × 10^4^ enriched C-kit^+^ HSCs were incubated with fluorescein isothiocyanate (FITC)-conjugated antibody C-kit (Santacruz Co., Dallas, USA) (1 μg/10^6^ cells) and subjected to FACS analysis. In addition, immunocytochemical (ICC) analysis was performed. The ICC protocol was described previously by Fathi et al. (2020) [26].

### 2.5 Co-culture of rat BM-resident C-kit^+^ HSCs and rat BM-derived MSCs

The co-culture procedure has been previously described by Fathi et al. (2022) [22]. Briefly, rat BM-derived-MSCs were plated into *trans*-well plates at 1 × 10^6^ cells/well in complete culture medium containing DMEM-low glucose and 10% FBS (Gibco Co., Carlsbad, USA). After 24 h, 1 × 10^6^ C-kit^+^ HSCs were seeded into two groups; culture of C-kit^+^ HSCs alone as the control group and co-cultured C-kit^+^ HSCs and BM-derived-MSCs as the experimental group. The granulocyte differentiation of C-kit^+^ HSCs were induced by 50 ng granulocyte colony stimulating factor (G-CSF) (Biolegend, Co., San Diego, CA, USA) in both control and experimental groups. After 7 days treatment cells were subjected to Western blot analysis for the intensity of granulocyte marker assessment.

The cells were divided into the two groups: control group (C-kit^+^ HSCs alone), and experimental group (co-cultured C-kit^+^ HSCs with MSCs).

### 2.6 Granulocyte markers and Wnt/β-catenin protein assessment

At the end of the co-culture time, granulocyte-differentiated C-kit + HSCs were collectedand proteins extracted. The equal protein amounts (50 μg) were subjected to the SDS-PAGE and transferred to polyvinylidene difluoride (PVDF) membranes. Next, membranes were incubated with primary antibodies β-actin (1:1000, Santacruz Co., Dallas, USA, sc-69879), CD34 (1:1000, Santacruz Co., Dallas, USA, sc-7324), CD16 (1:1000, Santacruz Co., Dallas, USA, sc-70548), CD11b (1:1000, Santacruz Co., Dallas, USA, sc-20050), CD18 (1:1000, Santacruz Co., Dallas, USA, sc-8420), Wnt (1:1000, Santacruz Co., Dallas, USA, sc-74537), and β-catenin (1:1000, Santacruz Co., Dallas, USA, sc-7963), and incubated with secondary antibodies (1:5000). Antigen-antibody complex were detected by enhanced chemiluminescence (ECL) reagent. β actin was used as the loading control [27,28].

### 2.7 Absolute telomere length (aTL) measurement

Following the 7 days co-culture, granulocyte differentiated C-kit^+^ HSCs were collected and the aTL was measured by real-time PCR as previously described by Fathi and Farahzadi (2022) [29]. For this purpose, two standard curves including telomere and single copy gene (SCG) standard curves were drawn as mentioned bellow (Primer sequences were listed in Table 1).

#### 2.7.1 Telomere standard curve

A telomere standard curve was created by the dilution of known quantities of a synthetic 84-mer oligonucleotide containing TTAGGG only. For generating a standard curve, the serial dilutions of TEL STD A (10^−1^ [1.18 × 10^8^] through to 10^−6^ [1.18 × 10^3^] dilution) were performed.

#### 2.7.2 Single copy gene (SCG) standard curve

SCG was used for determining the genome copies per sample. For generating a standard curve, the serial dilutions of SCG STD A (10^−1^ through to 10^−6^ dilution) were performed.

### 2.8 Cytokine measurement

Culture media were collected from both control and experimental groups and enzyme-linked immunosorbent assay (ELISA) was performed (R&D Systems, China). In brief, a 96-well plate was coated with detection Reagent A for 16 h at 4 °C. Then, cell culture media were added into the 96-well plate, which had been coated with IL-3 (Boster, Cat no: EK1324), IL-4 (R & D Systems, Cat no: DY504), and IL-6 (R & D Systems, Cat no: DY506-05) antibodies, and detected via the ELISA sandwich technique.

### 2.9 Statistical analysis

The results were analyzed using the software program Graph Pad Prism version 6.01. T-test and two-way ANOVA were used to determine the significant difference among groups.

## 3 Results

### 3.1 Culturing and characterization of rat BM-derived MSCs

**Fig. 1:**
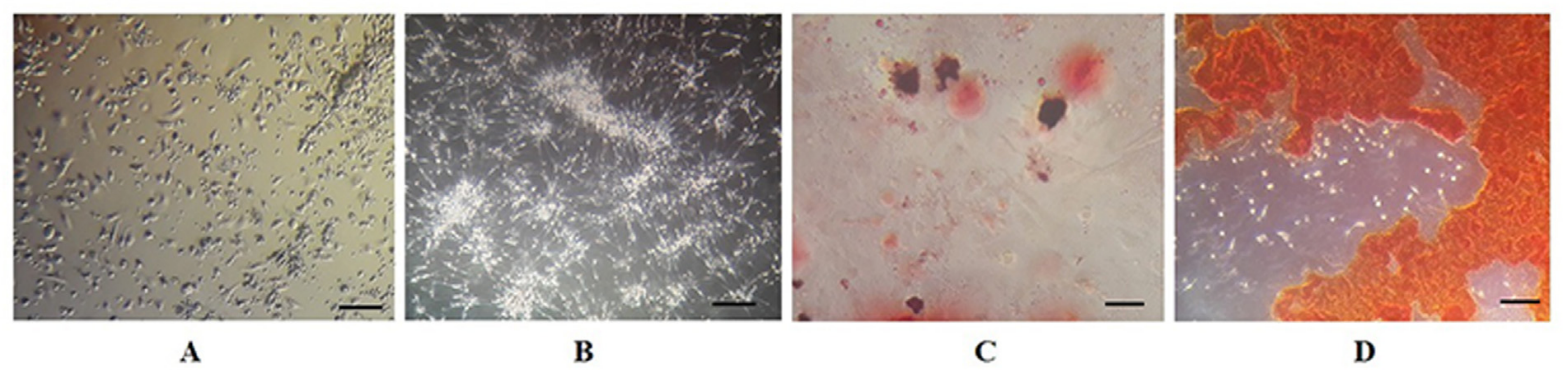
Morphological appearance of BM-derived MSCs. (A) Spindle-shaped appearance of BM cells at day 3, and (B) at day 7 (scale bar = 40X). **Multi-lineage differentiation of BM-derived MSCs**, (C) Oil-red O staining of lipid vacuoles were stained after adipogenesis (bar = 20 μm); (D) Alizarin red staining of mineralized cell aggregates at the end period of osteogenic differentiation (bar = 20 μm).

**Fig. 2:**
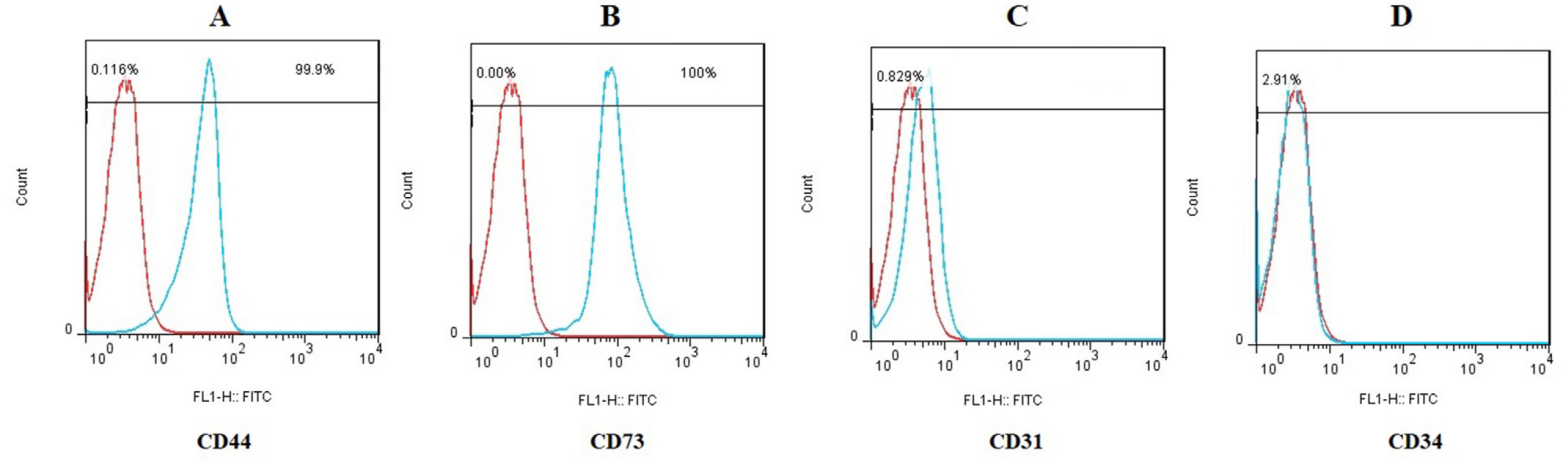
Phenotypical characterization of BM-derived MSCs by flow cytometry. (A) The BM-derived MSCs were positive for (A) CD44 (97.9%) and (B) CD73 (99.3%) and negative for (C) CD31 (0.20%) and (D) CD34 (0.47%). FlowJo software (version 6.2) was used for analyzing of flow cytometry data.

As shown in Fig. 1 A and B, rat BM derived-MSCs like other MSCs appear morphologically as spindle-shaped cells. The adipogenic and osteogenic multi-lineage differentiation of cells were documented in Fig. 1C and D. Next, the immunophenotypical characterization of cells by flow cytometry showed 97.9% CD44 and 99.3% CD73 expression of mesenchymal markers (Fig. 2A and B). However, the hematopoietic markers CD31 (0.20%) and CD34 (0.47%) were not expressed in rat BM derived-MSCs (Fig. 2C and D).

### 3.2 Identification of rat BM-resident C-kit^+^ HSCs

**Fig. 3:**
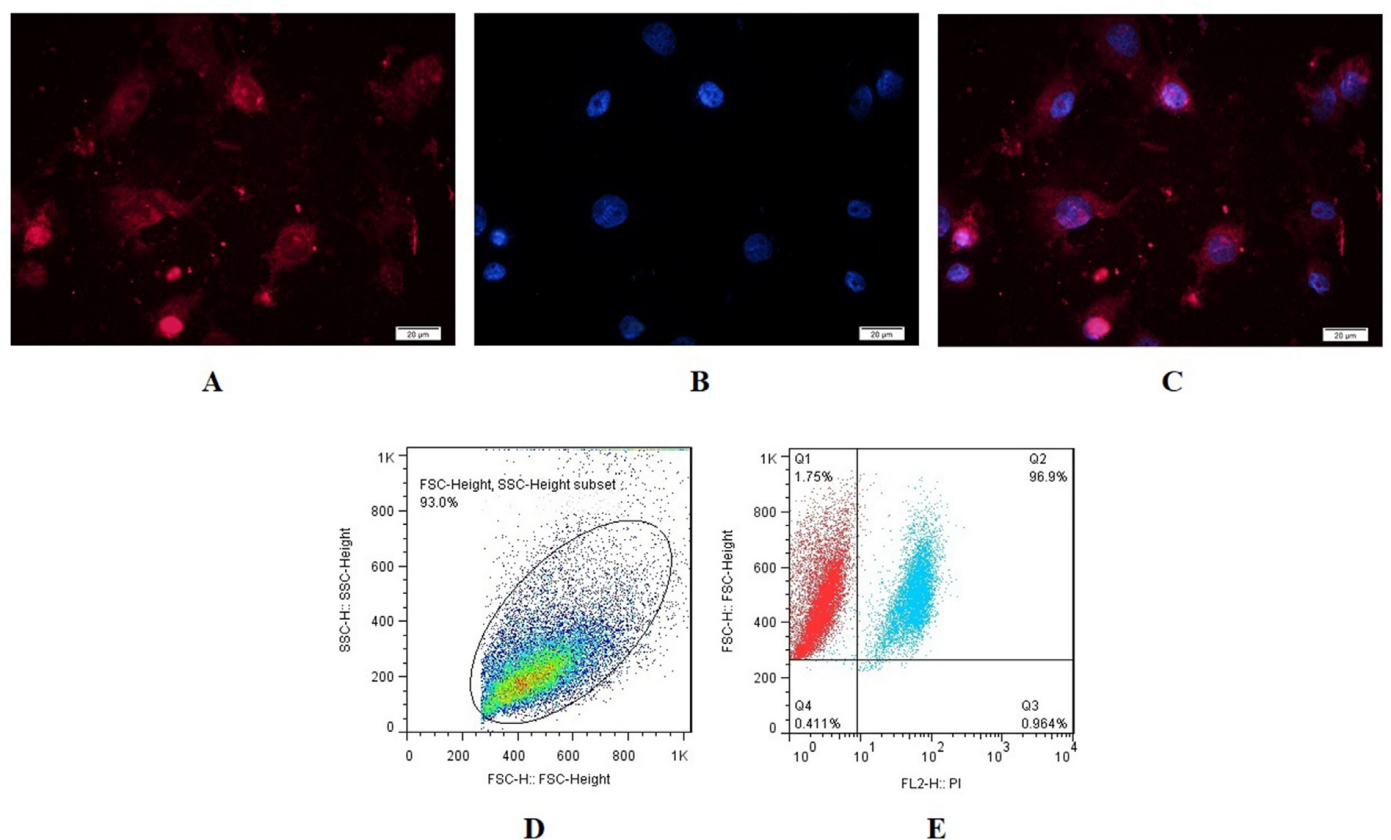
Characterization of BM-derived C-kit^+^ cells by ICC and flow cytometry analyses. (A–C) C-kit ^**+**^ cells were identified by immunofluorescence labeling; red = PE-conjugated C-kit; blue = DAPI; (size bar = 20 μm); (D) total population of cells used for C-kit evaluation; (E) flow cytometry: 96.9% CD117 positive cells; data presented as mean ± SD.

The purity of the rat BM-resident C-kit^+^ cells enrichment was assessed by ICC and flow cytometry. As shown in Fig. 3 A-C, the C-kit marker is shown by red fluorescence and DAPI staining of nuclei in blue. In addition, the flow cytometry analysis demonstrated that C-kit^+^ cells had high (96.9%) C-kit expression levels. Fig. 3D shows more detailed total cell population of cells. Fig. 3E shows the shift of C-kit^+^ cells (blue dots) from the isotype control population (red blots).

### 3.3 Western blot analyses

**Fig. 4:**
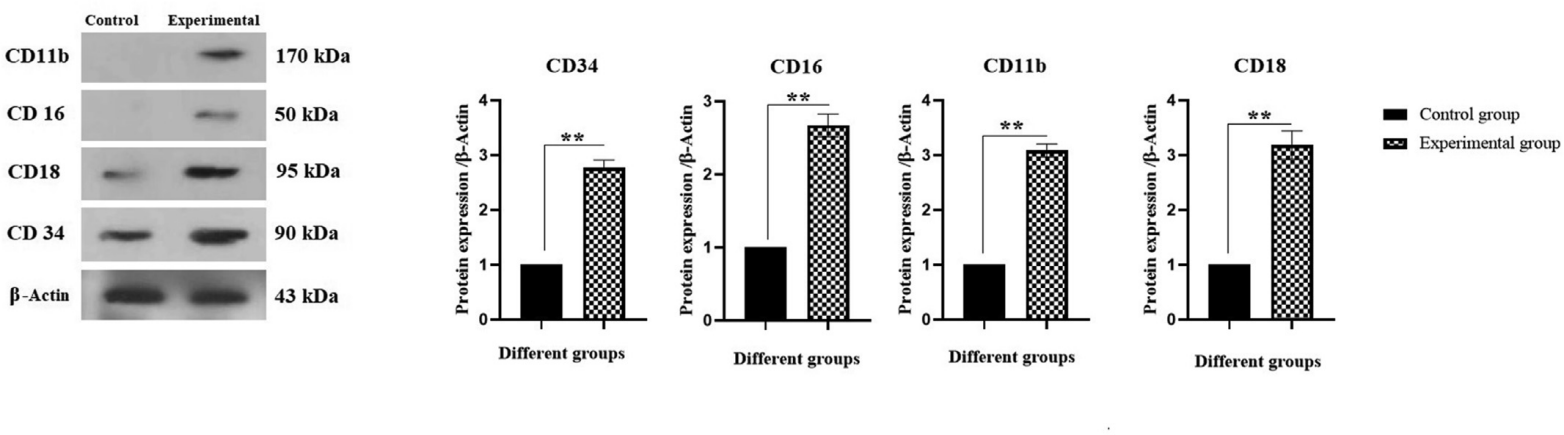
Effect of BM-derived MSCs on CD34, CD16, CD11b, and CD18 protein expression in granulocyte differentiated BM-derived C-kit^+^ cells. Culture of cells in plates and proteins lysates were extracted as appear in the Method section. The data was analyzed by Western blotting (∗∗*P* < 0.01).

**Fig. 5:**
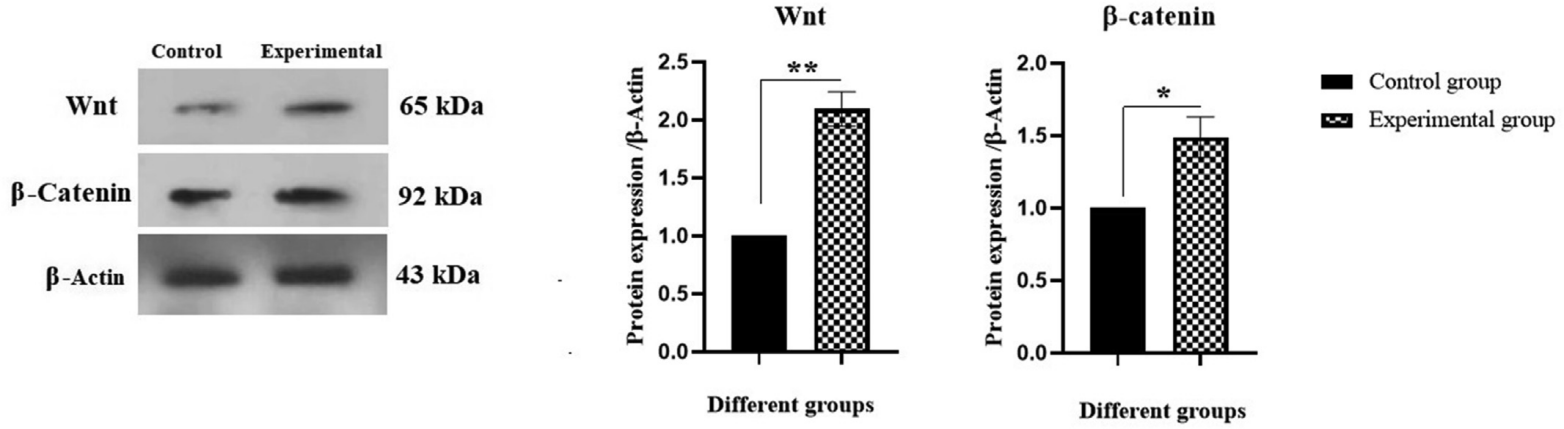
Effect of BM-derived MSCs on expression of Wnt and β-catenin in granulocyte differentiated, BM-derived C-kit^+^ cells. Culture of cells in plates and proteins lysates were extracted as appear in the Method section. The data was analyzed by Western blotting (∗∗*P* < 0.01 and ∗*P* < 0.05).

Following the co-culture, granulocyte differentiated C-kit^+^ HSCs were collected and granulocyte cell markers were analyzed by western blot as shown in Fig. 4. These results revealed that the expression levels of CD34, CD16, CD11b, and CD18 markers were significantly increased (2.77-, 2.67-, 3.08-, and 3.18-fold times, respectively) in the experimental group compared with the control group (∗∗*P* < 0.01). Also, the Wnt and β-catenin signaling pathways were investigated. As shown in Fig. 5, Wnt and β-catenin protein expression levels were significantly (2.09- and 1.49-times, respectively) increased in experimental group when compared to the control group (∗∗*P* < 0.01 and ∗*P* < 0.05, respectively).

### 3.4 Rat BM-derived MSCs increase the aTL of granulocyte differentiated rat BM-derived C-kit^+^ cells

**Fig. 6:**
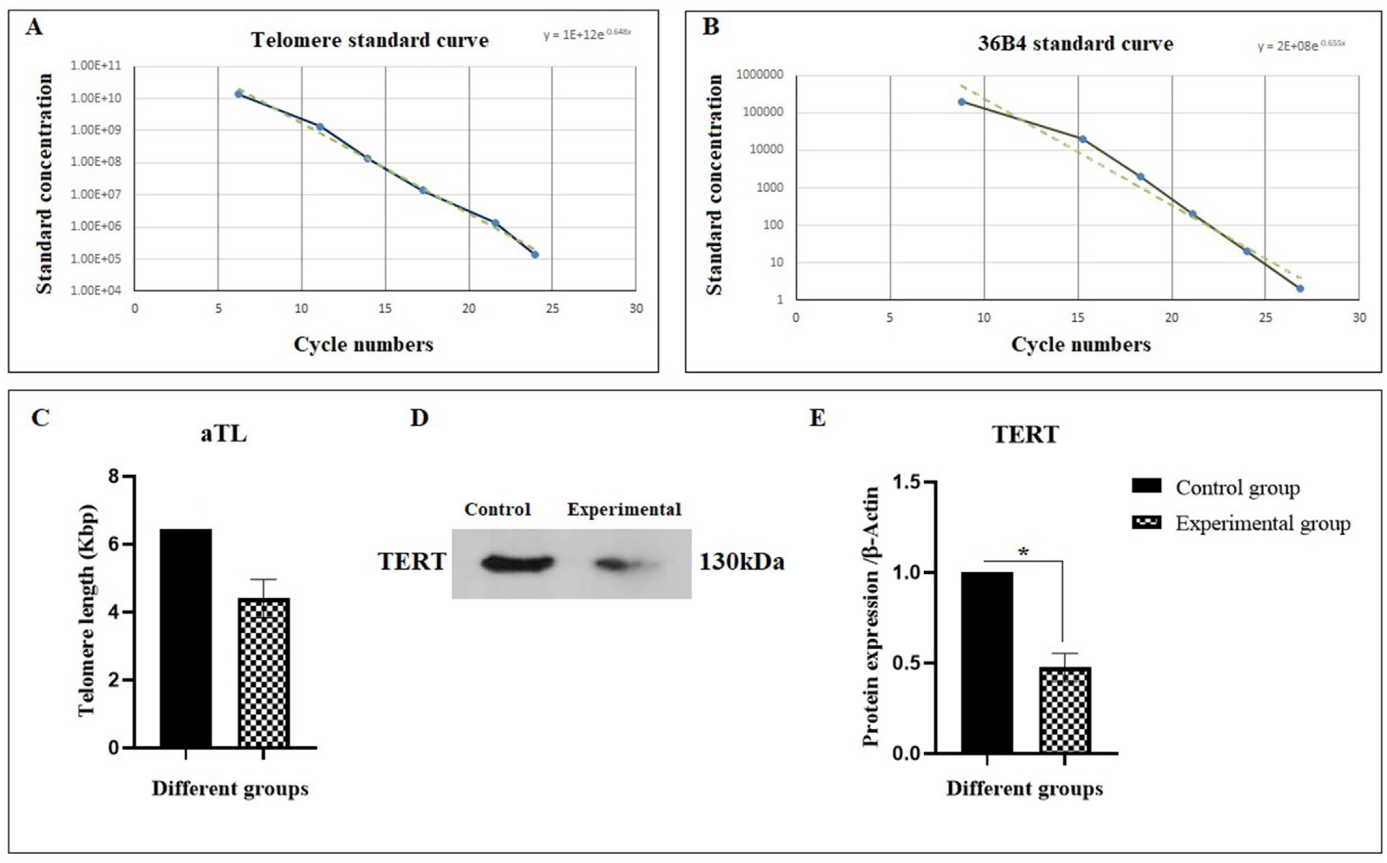
aTL measurement of granulocyte differentiated BM-derived C-kit^+^ cells. Graph (A) shows the telomere and (B) the 36B4 standard curve. (C) 20 ng/μl DNA samples were subjected to real-time PCR to evaluate aTL in triplicates. The data were analyzed as kb/reaction and the genome copies/reaction for the telomere and the SCG. (D) MSCs was significantly decreased the TERT protein expression of C-kit^+^ HSCs (∗∗*P* < 0.01; vice versa control, n = 3).

Quantitative real-time PCR was done for aTL measurement. As shown in Fig. 6A–C, the aTL was decreased in experimental group (4.4 kbp) in comparison with the control group (6.45 kbp). This finding demonstrated the role of MSCs in increasing the aTL of granulocyte differentiated rat BM-derived C-kit^+^ cells. Furthermore, the expression of TERT protein in the experimental group was significantly (0.47-fold) decreased when compared to the control group (Fig. 6D and E).

### 3.5 Cytokine secretion of granulocyte differentiated rat BM-derived C-kit^+^ cells

**Fig. 7:**
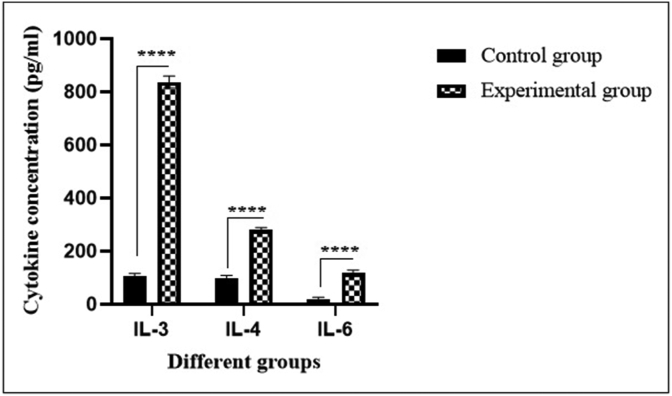
Cytokines secretion levels of IL-3, IL-4, and IL-6 from control and experimental groups (∗∗∗∗*P* < 0.0001, n = 3).

The ELISA measurements identified that rat BM-derived MSCs caused an increase in secretion of several cytokines (IL-3, IL-4, and IL-6) that may affect the granulocyte differentiation. These measurements revealed that the secretion of IL-3, IL-4, and IL-6 were significantly (∗*P* < 0.0001) increased in experimental group compared to the control group (Fig. 7). In particular, IL-3, IL-4, and IL-6 levels were increased about 5.34-, 2.88- and 6.42-folds in the culture media of the experimental group when compared to the control, respectively.

## 4 Discussion

Nowadays, stem cells including MSCs and HSCs definitely come into consideration in targeted tissues and organs systems. Co-culturing of HSCs with MSCs has received much attention in research since in the BM microenvironment niche, stromal cells have an effective role in providing a suitable network for many biochemical mediators as well as cytokines, e.g. stem cell factor (SCF) or CSFs that regulate hematopoiesis [30]. It was proven that co-culturing of HSCs with MSCs activates HSCs proliferation and maintains a primitive immune phenotype over a number of cell divisions. Proliferation of HSCs is enhanced by increasing cell passage number and MAPK1, N-cadherin, and vascular cell adhesion molecule 1 (VCAM1) concentrations. The activation of MAPK1 pathway is mediated by many growth factors including interleukin-3, stem cell factor, and erythropoietin [31]. Stem cells key properties such as multi-lineage differentiation, self-renewal, and tissue engineering are mainly affected by the soluble biochemical signals. MSCs produce many biochemical mediators and cytokines like IL-6, SCF, Flt3-L, TPO, CSFs (G-CSF, M-CSF, GM-CSF), that can affect BM derived-HSCs. They can also facilitate the differentiation of cells into the myeloid (monocytes/macrophage, granulocytes, and megakaryocyte) and lymphoid (T-cell, B-cell, and NK-cells) cell lines [32]. Even more, a previous study by Majumdar et al. (1998) reported that MSCs support hematopoiesis in long-term BM cultures [33]. UCB-MSCs promote granulocyte differentiation of APL-derived NB4 cell line and elicit an additive effect, when used in combination with all-trans vitamin A (ATRA) [16]. This promotion occurs by secreting interleukin-6 (IL-6) and activation of MAPK/ERK signaling pathway. This hypothesis was documented on one hand by the evidence that UCB-MSCs after co-culturing with APL-derived NB4 cells secrete a large amount of IL-6 and on the other by enhanced phosphorylation of MAPK/ERK, and inhibition of this signaling pathway [16]. Other mechanisms, such as the role of c-Myc in BM leukemogenesis have also been reported. It was proven that the expression of c-Myc in BM progenitors induces acute myelogenous leukemia (AML), while its inhibition induces cell cycle arrest and terminal differentiation of AML cells [34,35].

In the present study, the MSCs and C-kit^+^ HSCs were isolated from rat BM. After culturing and characterizing these cells, the interaction between MSCs and C-kit ^+^ HSCs was investigated under *in vitro* co-culture conditions. At the end of the co-culture period, the granulocyte differentiated rat BM-derived C-kit^+^ cells were collected and subjected to the Western blot analysis and real-time PCR for investigating the granulocyte marker expression, Wnt/β-catenin signaling components and aTL measurement, respectively. Accordingly, CD11b surface marker is expressed on the cell surface of monocytes and neutrophils. This surface marker provides the possibility of other factors to bind the granulocyte growth factors by specific ligands such as C3bi, fibrinogen, intracellular adhesion molecules-1 (ICAM-1), and Factor x. Additionally, it prepares neutrophil cells for phagocytosis and so leads monocyte/macrophage and T-cells to express immune responses to damages. The detection of CD11b in tissues and blood is an evidence supporting the proper function of immune T-cells and monocyte/macrophage cells in countering inflammation and viral attacks [36]. It has been observed that MSCs could selectively promote the expansion of CD11b^+^ cells from the BM.

Integrins mediate crucial adhesive recognitions through its ability in binding to multiple unrelated ligands^35^. CD16 is an integrin expressed on the surface of neutrophils, monocyte/macrophage, and a particular type of lymphocytes, i.e., NK cells. This surface marker determines the cell function and cellular distribution. CD16 presence on the neutrophil surface is a known maturity indicator and its absence is a sign of leukocytosis and tissue necrosis [37].

Yet another leukocyte integrin, CD18, consists of three heterodiameric *trans*-membrane receptors, named CDlla/CD18 (LFA-l), CDllb/CD18 (Macl and CDllb), and CD11c/CD18. These are broadly distributed on all leukocytes of lymphoid and myelomonocytic lineage.

CD34 is a surface glycol phosphoprotein expressed on progenitor cells, small-vessel endothelium, and embryonic fibroblasts. CD34^+^ cells include CFU-M, CFU-G, CFU-GM, BFU-E, which are particularly rich in the primary types of colony-forming cells such as CFU-mix and CFU-blast^36^. This maker as BM harvested hematopoietic cells is clinically used for transplantation and gene therapy studies. These cells have accelerated studies focused on the developmental hematopoiesis.

Flores-Guzman et al. in their study showed that rat BM-derived MSCs play a key role in primitive cell phenotype maintenance in CD34^+^/CD38^−^Lin^-^ populations [38]. Other studies indicated that rat BM-derived MSCs support HSCs expansion. Rasmusson et al. (2003) investigated the effect of MSCs on cytotoxic T lymphocytes (CTLs) and NK cells. Their findings showed that MSCs escaped from being detected by CTLs and all reactive NK cells also inhibited the formation of cytotoxic T-cells [39].

Emerging evidence generally supports the belief that cell differentiation often coincides with the activation or suppression of the signaling pathway. The importance of Wnt signaling in hematopoietic development has been reported previously. Activation of the Wnt pathway increased the number of HSCs as identified by colony forming cell assay [18,20].

In a study by Fathi et al. (2021), it was reported that co-culturing of MNCs with rat BM-derived MSCs cause an increase in the expression levels of granulocyte markers of MNCs, e.g., CD34, CD16, CD11b, and CD18 [23]. Based on these data, one can conclude that MSCs may affect the granulocyte differentiation of MNCs via ERK protein expression. Nikkhah et al. (2018) investigated the effect of MSCs on the granulocytic differentiation of HL-60 cells. This study showed that MSCs support the granulocytic differentiation of the human promyelocytic leukemia cell line HL-60 [17]. In the present study, the role of MSCs was investigated in the granulocytic differentiation of rat BM-derived C-kit^+^ cells and the results demonstrated that MSCs cause a significant increase in the expression levels of CD34, CD16, CD11b, and CD18 surface markers of rat BM-derived C-kit^+^ cells by about 2.77, 2.67, 3.08, and 3.18 folds, respectively. Likewise, in the previous study conducted by Fathi et al. (2021), it was indicated that MSCs cause to increase in the CD34, CD16, CD11b, and CD18 surface markers. In addition, in the study by Nikkhah et al. (2018), it was pointed that MSCs cause an increase in the CD11b surface marker as well as some granulocyte subset-specific genes such as PU.1, CD11b, lysozyme, MPO, CD64 that lead to an increase in the HL-60 leukemic cells [17]. As well, this study indicated that Wnt/β-catenin protein expression has significantly increased in the experimental group compared to the control group. In the current study, IL-3, IL-4, and IL-6 were evaluated using the ELISA. The results indicated that the secretions of IL-3, IL-4, and IL-6 have more increased in the experimental group when compared to the control group.

Accordingly, our results are in line with those of the previous studies regarding the important role of cytokines in the granulocyte differentiation of C-kit^+^ HSCs cells.

Further studies on molecular regulation of the granulocyte-differentiated C-kit^+^ HSCs will have to be undertaken to understand better these processes. TL shortening is known as one of the key characteristics of cell differentiation in cell therapy. Some studies have shown that aging is associated with the loss of TL in HSCs. In this study, our findings on aTL measurement of granulocyte-differentiated C-kit^+^ HSCs supported the previous findings of other investigations, showing that the differentiated cells have the shortest TL.

## Conclusion

In conclusion, our study for the first time demonstrated that rat BM-derived MSCs play an important role in promoting the granulocyte differentiation of rat BM-derived C-kit^+^ HSCs. This finding brings a new insight into the importance of Wnt/β-catenin signaling pathway during this differentiation process.

## Author's contribution

E.F. as the executive of the project had main contribution in conception and design, data analysis, and manuscript writing; I. V. as the main colleague of the project in providing the kits and some materials needed for this project as well as interpretation of experimental procedure; R.F. and S.A.M − N as colleagues involved in performance of experiments, data analysis and manuscript writing.

## Funding

This work has been supported by the Center for International Scientific Studies & Collaborations (CISSC), Ministry of Science Research and Technology of Iran.

## Ethics approval

The ethical code was obtained from the Committee on the Ethics of Tabriz University of Medical Sciences (Ethics No. IR. TBZMED.VCR.REC.1397.322).

## Statement of human and animal rights

All of the experimental procedures involving animals were conducted in accordance with the ethics committee of Tabriz University of Medical Sciences (IR.TBZMED.VCR.REC.1397.322) approved protocols. This article does not contain any studies with human subjects.

## Statement of informed consent

There are no human subjects in this article and informed consent is not applicable.

## Availability of data and materials

The data sets used and/or analyzed during the current study are available from the corresponding author on reasonable request.

## Declaration of competing interest

The authors declare that they have no conflict of interest.
